# The SASOP/PsychMg guidelines for psychiatric independent medical examinations

**DOI:** 10.4102/sajpsychiatry.v30i0.2187

**Published:** 2024-04-04

**Authors:** Renata Schoeman, Antoinette L. Miric, Mvuyiso Talatala, Christoffel Grobler

**Affiliations:** 1 Department of Leadership, Stellenbosch Business School, University of Stellenbosch, Bellville, South Africa; 2 Department of Family Medicine and Primary Care, School of Clinical Medicine, University of the Witwatersrand, Johannesburg, South Africa; 3 Department of Psychiatry, Faculty of Health Sciences, University of the Witwatersrand, Johannesburg, South Africa; 4 Department of Public Health and Health Systems, Faculty of Health Sciences, University of Pretoria, Pretoria, South Africa; 5 Department of Psychiatry, Faculty of Health Sciences, Walter Sisulu University, Port Elizabeth, South Africa

These guidelines, developed by the South African Society of Psychiatrists (SASOP) and the Psychiatry Management Group (PsychMg) offer a comprehensive framework for conducting psychiatric independent medical examinations (IMEs) in South Africa. They serve as a valuable resource for psychiatrists involved in evaluating disability claims, providing a standardised approach to psychiatric assessment and reporting. These guidelines address the challenges inherent in determining psychiatric impairment, including premature determinations of permanent inability to work and inconsistencies in diagnosis and prognosis. They emphasise the importance of objectivity, avoiding conflicts of interest and maintaining professional ethics in the IME process. By providing a structured methodology and reporting format, these guidelines aim to facilitate informed decision-making by third parties, such as insurance companies and the courts, in assessing mental health-related disability claims.

## Disclaimer

These guidelines do not aim to provide an exhaustive review of the relevant literature constituting the evidence base. It is the responsibility of practitioners to maintain a high level of personal knowledge and expertise. Despite the availability of numerous evidence-based treatment guidelines worldwide, access to healthcare and treatment remains limited for many patients in South Africa. Common mental health disorders are often poorly identified and treated at the primary healthcare level, and access to specialist resources is restricted. Many practitioners are faced with the shortcoming of not being adequately trained for conducting psychiatric IMEs – yet, are being expected to undertake such evaluations. These guidelines should not be considered as a policy document, but as an aid in management and gold standard practice of psychiatric IMEs.

## The process

South African Society of Psychiatrists established the disability task team in 2020, with Prof C.G as the convenor. The task team’s primary objective is to update the previous guidelines of 2017 to align with advancements in the field and *The Diagnostic and Statistical Manual of Mental Disorders, Fifth Edition (DSM-5)* nomenclature.^1,2^ Achieving this goal requires a collective and dedicated effort from individuals with a special interest in and passion for conducting IMEs of psychiatric impairment. The aim is to enhance knowledge and ensure consistency in approach throughout South Africa while providing the insurance industry and the courts with a professional standard for psychiatric IMEs in the country.

Grobler, supported by the task team, was entrusted with drafting the guidelines. Subsequently, several virtual meetings were conducted to discuss different drafts of the document. The guidelines were shared via email with task team members to gather written feedback and evidence-based suggestions, which were then incorporated into the guidelines. The final version of the guidelines underwent a round of written approval from task team members before being formally approved by the task team. The guidelines were submitted to the SASOP and PsychMG boards for recommendation and ratification.

## Preface to the fourth edition

The SASOP formed a task team in 1995 to address the need for a standardised approach to examining patients with mental health disorders for disability assessment purposes. From the beginning, SASOP embraced an approach that was rooted in the methodology and guiding principles of psychiatric impairment assessment, as outlined in the American Medical Association’s (AMA) Guides to the Evaluation of Permanent Impairment.^3^ The initial draft of the SASOP guidelines was published in 1996.^1^

Subsequently, the second edition of the guidelines was developed, extensively reviewed and approved by the SASOP executive committee.^4^ It was published in 2001 and was based on the fifth edition of the AMA Guides to the Evaluation of Permanent Impairment.^5^ The second edition of the guidelines^4^ provided criteria for evaluating psychiatric disorders, determining the degree of impairment, identifying permanence and offering a suggested format for psychiatric reports. They also covered common psychiatric disorders associated with disability claims.

In 2017, the third edition of the guidelines incorporated advancements in diagnosing and managing psychiatric disorders. Notably, the third edition introduced a disability prevention model, emphasising the significance of maintaining employment for as long as possible.^6^

In April 2021, Chapter 14 of the AMA Guides, which focuses on mental and behavioural disorders, underwent revisions in alignment with changes in DSM-5.^7^ As a result, it became necessary to update the SASOP Guidelines to the Assessment of Psychiatric Impairment and rename it to the SASOP/PsychMg Guidelines to Psychiatric IMEs in order to reflect these changes and determine an appropriate methodology for rating psychiatric impairment in South Africa.

## Introduction

Since the onset of the coronavirus disease 2019 (COVID-19) pandemic, there has been a significant increase in claims for occupational mental health disability, with no indication of this trend declining anytime soon.^8^ While the physical health impact of the pandemic appears to be subsiding, the mental and behavioural health consequences may just be emerging.^8^

An IME is a voluntary assessment that is typically conducted only once for an individual. It involves gathering information from various sources and is carried out by a licensed healthcare professional who does not have a treating relationship with the person being evaluated. It is important to avoid using the term ‘patient’ when referring to the examinee to prevent any confusion with a conventional doctor–patient relationship, which is not permitted during an IME.^8^

Ethical and professional standards preclude the treating health professional from conducting an IME or giving an opinion on a forensic matter of a patient under the professional’s care.^9,10^ Psychiatrists are frequently called upon to conduct IMEs for various purposes, often at the behest of third parties such as insurance companies, lawyers and the court. These assessments are crucial in evaluating disability claims and providing valuable insights and expert opinions that are relied upon by these parties to make informed decisions.^11^

These guidelines aim to provide South African psychiatrists with a comprehensive method for assessing and reporting on psychiatric impairment, which may assist adjudicators in deciding on disability claims or financial compensation for their clients.

## Ongoing challenges in the determination of psychiatric impairment

The goal of insurers and the courts is to make sound decisions regarding legitimate disability claims.

The disability task team has identified some ongoing challenges in different aspects of psychiatric impairment in South Africa, which may hamper this goal. These include:

- Prematurely determining permanent inability to work without allowing sufficient time for treatment to take effect.
- Inconsistencies in diagnosis, management and determination of prognosis among medical professionals.
- Attempting a return to work without adequate occupational therapy intervention.
- Declaring a permanent impairment despite inadequate psychopharmacological management, multidisciplinary referrals or appropriate rehabilitation.
- Lack of objectivity in the reports.
- Lack of standardised reporting formats.
- Lack of a standardised approach to assessing psychiatric impairment.
- A heavy reliance on self-reported information from the examinee.
- Offering opinions without incorporating a reasonable degree of objective findings and a diagnosis of a mental disorder.

Assessing occupational impairment can be more challenging when mental illness is subtle and combined with personality factors or potential secondary gain. It is important to emphasise that the presence of a diagnosis does not necessarily imply impairment. Functional impairment can vary widely among individuals with the same psychiatric disorder, and many factors interact to determine an individual’s functioning in a specific work situation.

Independent medical examination providers are advised to adhere to a minimum standard of introducing objectivity into the IME process.^8^ As stated, the treating psychiatrist should avoid serving as an independent medical examiner on behalf of their patients, as this dual role can be detrimental to the therapeutic relationship. Both treating and independent psychiatrists are encouraged to engage in respectful and professional discussions about examinees, avoiding an adversarial approach and respecting each other’s roles in the process of assessing and managing impairment.

## Aims of these guidelines

These guidelines will assist psychiatrists in conducting IMEs and with writing comprehensive reports, providing a standardised method for psychiatric evaluation and reporting. This includes a comprehensive list of essential psychiatric information required by third parties to make informed decisions about disability claims.

## Disability prevention and the importance of remaining in employment

The third edition^6^ of these guidelines emphasised a shift towards recovery, adopting a disability prevention model and recognising the importance of remaining in employment. These principles continue to be central in this current guidelins, which are listed as follows:

- All mental healthcare practitioners, including treating psychiatrists, psychologists and occupational therapists, should focus on enhancing resilience and promoting early return to work.
- Early involvement of occupational therapy services is essential for functional capacity assessment, vocational rehabilitation and collaboration with the employer.
- Treating psychiatrists should not lead patients to believe that they will be declared permanently unfit based solely on a psychiatric report.
- The independent psychiatrist’s role is to assess the examinee and rate areas of impairment, indicating whether the impairments are permanent or not, while the decision regarding disability lies with the third party.

## The role of occupational therapists and a return-to-work plan

Occupational therapists play a crucial role in the work rehabilitation process. They contribute through job analysis and functional capacity assessments and by facilitating the reintegration of workers with injuries or disabilities into the workforce. Job analysis involves assessing a job’s physical, mental and environmental aspects to ensure compatibility with the employees’ abilities. Functional capacity assessments help to determine the residual abilities of injured workers, providing essential data for evaluating their capacity to return to work.^11^

Vocational rehabilitation serves as a pathway for impaired individuals affected by illness or disability, whether temporary or permanent, to regain employment or engage in productive activities. Involving occupational therapists early in the sick leave process helps bridge the gap between employers and patients, facilitating a successful return to work.^11^

Treating psychiatrists should promptly implement a return-to-work plan when recommending sick leave for 1 month or more. This plan should align with a recovery-focused model to prevent permanent disability. Prolonged absences from work can lead to disability, underscoring the importance of early interventions and well-structured strategies for reintegration.^12^

Once a patient is granted extended sick leave, the potential risk of permanent disability should be considered. Permanent disability is associated with increased mortality rates and diminished quality of life in various domains. Every effort should be made to prevent the onset of permanent disability.^13^

## Definitions

### Impairment

Impairment refers to a ‘significant deviation, loss or loss of use of any body structure or body function in an individual with a health condition, disorder or disease’.^5^ Assessing impairment involves making a diagnosis and considering treatment options to determine which functions persons are still able to perform and which are not.

An impairment can only be considered to be permanent if the following conditions are met^13^:

- Optimal treatment has been administered for a reasonable period.
- Treatment has followed known principles of evidence-based medicine.
- The natural course of the illness is known to lead to deterioration and continued impairment.
- The examinee has been fully compliant with treatment.

### Impairment rating

Impairment rating is a percentage estimate derived through consensus. It quantifies the loss of activity attributable to a particular health condition, considering the severity of the condition and the extent of its impact on individuals’ daily living activities. This metric allows physicians to provide a numerical assessment of the losses that persons experience as a result of their health condition, disorder or disease.^3^ Although rating impairment is not strictly part of an IME for insurance purposes, it is useful as it provides a benchmark for current impairment and assists in deciding on maximum medical improvement.

### Disability

Disability is defined as ‘activity limitation and/or participation restrictions in an individual with a health condition, disorder or disease’.^3^

In the insurance context, the extent of persons’ impairment will be considered in conjunction with their job description, policy disability clause conditions and personal factors – such as education, experience and age – to assess disability. Hence, in this context, disability assessment is a decision taken by a panel of experts, including a medical advisor, a legal advisor and a claims consultant.

### Maximum medical improvement

The AMA Guidelines indicate that maximum medical improvement refers to a permanent condition in which significant change is not anticipated over the subsequent 12 months.^2^ Maximum medical improvement represents the best achievable condition through all reasonable available medical treatment and may necessitate ongoing follow-up evaluations and interventions for optimal maintenance and relapse prevention.^3^

Permanency refers to a stable or unchanging impairment, with or without medical treatment, that is unlikely to improve in the future within the bounds of medical probability. Impairment ratings are conducted when a condition has reached a state of permanency. The term ‘maximum medical improvement’ is often used interchangeably with permanency.^3^

## Impairment, disability and maximum medical improvement

Assessing impairment involves a comprehensive review of the diagnosis and available treatment options. This enables a medically grounded determination of individuals’ functional abilities. Insurers and courts often rely on psychiatrists to provide professional opinions on whether the impairment is likely to be permanent and/or if the patients have reached maximum medical improvement.^3^

When considering maximum medical improvement or permanency, it is essential to acknowledge that certain mental health disorders may improve over time, and impairment may decrease over an extended period. While a decision on maximum medical improvement is taken when no significant improvement is expected to take place in the next 12 months, a diagnosis of permanent impairment is a more difficult decision to take. When permanent impairment is considered, an examinee may have to be reviewed after 2 or 3 years, as it is known that certain mental health disorders may improve over time and that the impairment may lessen over an extended period in some instances.^3^

The significant role of occupational therapists in evaluating functional impairments in patients with mental health disorders should be emphasised. Their input is crucial before final determinations are made regarding maximum medical improvement and permanent impairment.

In the context of impairment, disability and maximum medical improvement, it is important to remember the following^6^:

- Subjective distress does not equate to functional impairment.
- Employment dissatisfaction and psychosocial stressors do not necessarily indicate functional impairment.
- Psychiatric assessment relies heavily on the accuracy and completeness of patients’ self-reporting and the objectivity of information provided by the treating psychiatrist. Distortions, intentional or unintentional, may occur when compensation is a factor.
- It is crucial to note that a medical or psychiatric condition cannot be considered treatment-resistant, permanent or irreversible unless all reasonable and recognised treatment options have been explored.

In the South African context, it is essential to consult recognised treatment guidelines, such as those published by SASOP and the Standard Treatment Guidelines and Essential Medicines List for South Africa.^14,15^ Unfortunately, in a resource-constrained country like South Africa, most of the population cannot access optimal treatment. Even among those who can afford private healthcare, various factors, as identified by the task team, hinder the attainment of optimal treatment, including limited benefit access, exhausted benefits, waiting periods before claim payouts and the discontinuation of income and medical aid benefits before the start of claim payouts. These obstacles can have a significant negative impact on the accessibility and affordibility of psychiatric treatment.

While these constraints to adequate treatment are generally acknowledged, extensive discussion among the task team members leads to the recommendation that evidence-based treatment should be considered as the benchmark for optimal treatment. This includes psychopharmacological treatment, psychotherapy administered by qualified clinical or counselling psychologists and occupational therapy involving vocational rehabilitation. The level of care recommended should be comparable to that available in a tertiary academic psychiatric hospital in South Africa.

## General principles of an independent medical examination

Independent medical examinations are voluntary assessments conducted by licensed healthcare providers who are not responsible for the care of the examinees. These evaluations are performed for medicolegal and insurance purposes, and they rely on various sources of information to gain a comprehensive understanding of individuals’ conditions. It is important to note that the term ‘patient’ should not be used to refer to the examinee, as it could imply the existence of a traditional doctor–patient relationship, which is strictly prohibited during the IME process.^8^

Psychiatric IMEs can occur in various settings and focus on assessing the presence and impact of mental health disorders. The specific scope of IMEs is shaped by requesting parties’ medical information needs, and IME providers may be asked to provide insights into a range of relevant questions for the cases at hand.^8^

Psychiatric IMEs differ from clinical consultations in significant ways. One crucial difference is the absence of a traditional doctor–patient relationship during IMEs, and a limited duty of care is established that does not go beyond the obligation to perform unbiased evaluations. In IMEs, examiners must maintain neutrality and objectivity. Unlike in traditional treatment settings, where treating professionals advocate on behalf of patients, IME examiners must avoid assuming both roles (the so-called dual-agency conflict). Examiners are held to the same high standards and ethical obligations as in clinical consultations, with the expectation that they too will abide by the Health Professions Council of South Africa’s Ethical Guidelines for Healthcare Professions.^16^

To conduct psychiatric IMEs, evaluating psychiatrists should be registered with the Health Professions Council of South Africa, and independent medical examiners should possess the necessary experience, knowledge and skills to perform an IME efficiently. Despite the absence of explicit statutory requirements in South Africa for additional qualifications or special credentials for those conducting IMEs, it is highly recommended that health professionals actively pursue continuing education and training initiatives, such as the Evaluation of Permanent Medical Impairment Rating (based on the sixth edition of the AMA) short course. These courses familiarise psychiatrists with the methodology for assessing impairments using the AMA sixth edition guidelines.^3^ Ongoing education and peer support are provided by organisations such as the International Academy of Independent Medical Evaluators (IAIME), the American Board of Independent Medical Examiners (ABIME) and the Canadian Society of Medical Evaluators (CSME).^8,17,18,19,20^

Although empirical studies are limited, no evidence suggests that a virtual psychiatric IME is less valid than an in-person evaluation, assuming that the applied methodology is comparable, and the testing process has been validated for remote administration of the IME.^8^ With the rise in virtual consultations, it is prudent for independent medical examiners to confer with their indemnifying body regarding the feasibility of conducting IMEs outside South Africa, for example, in countries where they are not professionally registered.

## The independent medical examination process

Before conducting IMEs, requesting parties should provide relevant background information to examiners. It is highly recommended that examiners review the medical records before the evaluation to clarify any inconsistencies between examinees’ reported history and the information obtained from the records. If the submitted information is incomplete or insufficient, examiners should request additional information from referring parties.^8^

Having a chaperone present during a psychiatric IME is advisable to enhance examinees’ comfort, particularly in the cases of clients with past trauma or during physical examinations. If other parties, such as examinees’ legal counsel or designate, request to be present during the examination, the examiners have the right to decline to conduct the IME.^8^

Before starting IMEs, examiners should verify examinees’ identities by inspecting government-issued picture identification, such as a driver’s licence, identity document or a passport.

Ensuring valid consent is crucial during an IME. Examiners should explain the process clearly to examinees, using language they can understand, while considering any cognitive limitations. Examinees’ confirmation of understanding should be obtained, and their signature on the consent form signifies their agreement. It is essential to clarify that the IME is conducted for a specific medical issue and context, distinct from overall healthcare service delivery. Consent should be voluntary, informed and obtained without coercion.

It is important to explain to the examinee that the purpose of the examination is to write a report that will be sent to a third party, and therefore the traditional doctor–patient confidentiality does not apply. However, it is prudent to reassure the examinee that every attempt will be made to protect the dignity of the examinee in the choice of wording for the report.

In the case of examinees being unable to provide consent owing to impaired capacity, consent to perform the evaluation should be sought from the legal guardian or curator. If examinees are unwilling to provide informed consent, the IME should be terminated immediately, with notification given to the requesting parties. This upholds ethical principles and protects the rights of the examinees.^8^

Examinees should be urged to report any significant distress during the IME, and the examiners should promptly pause the evaluation, if necessary. In cases of severe distress, the IME should be halted and rescheduled.

To ensure a successful IME, sufficient time should be allocated for the assessment process. Avoiding disrespectful or aggressive comments or behaviour during the interaction is crucial, as individuals with mental health disorders may be particularly sensitive to such attitudes. Treating the examinees with respect and dignity, without providing therapeutic intervention, is of utmost importance. Privacy should be prioritised, and the IME’s environment itself should be comfortable and reasonably stress free.^8^

When performing an IME for an insurance claim, it should be explained to examinees that, although they have a right to their personal records, the report based on the IME belongs to the insurer who has requested the report. The examinee has the right to request a copy of the report from the insurer that requested the IME. It is important to caution examinees that they should be judicious about sharing the report owing to the sensitive nature of the contents thereof.

A comprehensive assessment is conducted during a psychiatric IME evaluation, including eliciting a history, reviewing records and performing a mental status examination. The mental status examination describes various aspects, such as general appearance and behaviour, mood, affect, speech, thought processes, thought content, perceptions, cognition, insight and judgement.

In addition to bedside tests, such as the Montreal Cognitive Assessment (MoCA),^21^ the Mini-Mental State Examination (MMSE)^22^ and the Saint Louis University Mental Status (SLUMS),^23^ a more thorough neuropsychological evaluation may be necessary in certain situations.

Despite the wide range of available psychological tests and their potential utility in helping to form opinions, the patient interview, review of records and mental status examination remain the foundation for evaluating the patient and determining the impairment rating. Neuropsychological testing is most useful in patients with subtle organic deficits – not obvious ones, where office assessment is often adequate.

The physical examination is typically not routinely conducted as part of a psychiatric IME and requires prior consent from the examinee, with a clear explanation provided for the need of a physical examination.

With the examinee’s permission, it may be necessary to gather collateral information from treating healthcare providers, family members or significant others who have firsthand knowledge of the examinee’s medical condition, its severity and functional impairment in various areas.

Before concluding IMEs, it may be helpful to ask the examinees if any further information could provide a more comprehensive understanding of the issues being assessed. This request should allow for an open-ended discussion, with sufficient time for examinees to share any additional relevant details. Examinees should be thanked for their time and cooperation. Examinees should be reminded that the reports will be sent directly to the requesting party and will not be shared with others, including the examinees’ healthcare providers or employers.^8^

## Specific recommendations for a psychiatric independent medical examination

Several recommendations for conducting a psychiatric IME are captured in Table 1.

**TABLE 1:** Recommendations for a psychiatric independent medical examination.

No.	Recommendation
1	Screen for past and current substance abuse, as this abuse can resemble symptoms of other psychiatric diagnoses.
2	Evaluate the individual’s legal history, including prior lawsuits, work-related injuries, driving under the influence, incarcerations and restraining orders.
3	Note any pattern of symptom overendorsement during the psychiatric interview.
4	Assess treatment and the response thereto.
5	Assess the examinee’s motivation to return to work and consider how the disease process may affect motivation (whether unconscious or conscious), if needs are being gratified, and whether signs of secondary gain are present.
6	Determine whether symptom exaggeration or malingering is present, which can range from subtle to overt.
7	Inquire about patient attitude towards the third-party payer (e.g. employer, insurance company) and the perception of the appropriate response to the situation.
8	Assess the influence of the litigation process on return to work and identify any history of failed attempts to return to work.

*Source*: Adapted from American Medical Association guides to the evaluation of permanent impairment. 6th ed. Rondinelli RD, Genovese E, Katz RT, et al., editors. Chicago, IL: American Medical Association; 2008.

No, number.

### Assessing treatment response

Evaluate the overall management (biopsychosocial) or pharmacological and/or nonpharmacological:

1. General:
  - Adherence to standard guidelines: Does the treatment align with accepted practices and procedures?
  - Evaluation of the treatment duration: Has the patient completed an adequate course of treatment?
  - Variety of attempted treatment options: Has an adequate number of diverse treatment options been explored and attempted?
  - Patient cooperation and medication adherence: Has the patient’s adherence to medication been evaluated, and has the patient cooperated with the treatment interventions? Note that certain mental health conditions may lead to diminished insight, which could potentially obstruct treatment.
  - Coexisting disorders: Are there any comorbid substance abuse issues or physical disorders causing mental symptoms, and have these been adequately addressed?
2. Pharmacological:
  - Has the patient’s adherence to medication been evaluated?
  - Are there any residual psychiatric symptoms?
  - Are there any side effects of medication?
  - Has the medication management been optimised for the patient’s specific condition?
3. Nonpharmacological:
  - Have appropriate referrals been made to psychologists, occupational therapists and social workers?
  - Has the frequency of these interventions been sufficient?
  - Are there barriers to obtaining these interventions?

### Motivation

Assessing individuals’ motivation is crucial, as it can have a significant impact on their ability to lead a productive life. Motivation can be influenced by various factors, including negative symptoms associated with psychiatric illness, medication side effects, fear of losing entitlements or benefits, workplace conflict, legal issues, personality and coping styles, demoralisation, social network support, substance use and neurological or medical conditions affecting cognitive function.^8^

### Malingering

Malingering refers to the intentionally feigning or exaggeration of symptoms for secondary gain. Examiners need to be vigilant about the possibility of malingering, especially in contexts that involve legal or financial incentives. Evaluators should examine the person’s history, physical and mental status and available information to identify any inconsistencies.

The use of the term ‘malingering’ may lead to criticisms of the examiner by administrators or decision-makers in the legal system. As it implies intentional deception by the patient, the term should be used carefully. The use of alternative terms such as ‘symptom exaggeration’ or ‘inconsistencies in symptom reporting’ should be considered as they are more precise and less contentious when a psychiatric disorder cannot be identified.^3^

### Diagnostic nomenclature

In South Africa, the DSM-5 is used for diagnosing mental health disorders.^2^ The release of the DSM-5-TR introduced updates to nomenclature and classification.^24^ While the DSM-5 was not specifically developed for forensic purposes, it is frequently used in this context, and the most current edition (DSM-5-TR) is expected to be applied. It is important for the examiner to adhere to the current criteria and avoid introducing personal or idiosyncratic diagnoses.^8^

The current task team reached a consensus to adopt a hybrid system: combining the diagnostic criteria and terminology of DSM-5-TR,^24^ with the five-axis system following DSM-4-TR^25^ guidelines for making a diagnosis.

### Rating psychiatric impairment

The methodology for rating psychiatric impairment recommended by SASOP follows the guidelines outlined in Chapter 14: Mental and Behavioral Disorders of the AMA Guides to the Evaluation of Permanent Impairment. This chapter provides a framework for evaluating impairments resulting from mental and behavioural disorders and utilises three scales: the Brief Psychiatric Rating Scale (BPRS),^26^ the Global Assessment of Functioning (GAF)^26^ and the Psychiatric Impairment Rating Scale (PIRS).^27^

Impairment rating using the AMA Guides is limited to certain diagnoses typically encountered in medicolegal settings: mood disorders (depressive disorders and bipolar and related disorders), anxiety disorders, obsessive-compulsive disorder, trauma- and stressor-related disorder and psychotic disorders (schizophrenia spectrum and other psychotic disorders). However, not all disorders are included in the AMA Guides as ‘ratable conditions’, that is, psychiatric reaction to pain (as the impairment rating for physical conditions already accounts for associated pain), substance intoxication and withdrawal, sleep disorders, dementia and delirium, intellectual disability and psychiatric manifestations of traumatic brain injury.^18^

### Impairment rating

**FIGURE 1:**
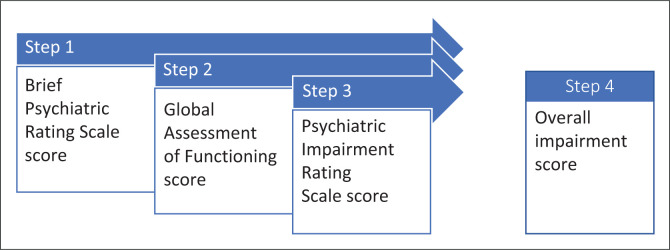
Steps in impairment rating.

The impairment rating for mental and behavioural disorders involves several steps to determine the scores for the BPRS, GAF and PIRS (Figure 1). The final impairment rating is the median value of these scores.^3^

#### Brief Psychiatric Rating Scale score

In the evaluation of mental health disorders, the BPRS^26^ is used to assess significant psychotic and nonpsychotic symptoms. This scale has demonstrated reliability in clinical trials and applies to both adult inpatients and outpatients.

The BPRS consists of 24 symptom constructs, each rated on a 7-point scale measuring severity. The scores range from ‘not present’ to ‘extremely severe’, using numbers 1–7. Items 1–14 are based on the individual’s self-report, while items 7, 12 and 13 also consider observed behaviour. Items 15–24 are rated on the basis of observed behaviour and speech. The total score is obtained by summing the scores of all 24 symptom constructs. Detailed instructions provided in the AMA Guides should be followed for accurate scoring.^3^

#### Global Assessment of Functioning score

The GAF^28^ is a 100-point rating scale designed to assess overall symptoms, occupational functioning and social engagement. The GAF score focuses solely on psychological, social and occupational functioning. It excludes impairments related to physical or environmental limitations. The GAF score is determined on the basis of an assessment of overall functioning in these domains.

#### Psychiatric Impairment Rating Scale score

The PIRS,^27^ modified for use in the AMA Guides, evaluates the behavioural consequences of psychiatric disorders on six scales that assess functional impairment in specific areas.

The PIRS score is determined by grading the patient from 1 to 5 in six domains:

- Self-care, personal hygiene and activities of daily living;
- Role functioning, social and recreational activities;
- Travel;
- Interpersonal relationships;
- Concentration, persistence and pace;
- Resilience and employability.

Scores for each domain are obtained from the relevant tables in the AMA Guides.^3^

The rating process involves the following steps:

- Assign a score from 1 to 5 to the patient for each of the six impairment domains.
- Arrange the six scores in ascending order (e.g. 1, 2, 2, 4, 4, 5).
- Identify the middle two scores from the arrangement of six scores. In the given example, the middle two scores are 2 and 4.
- Add the middle two scores together. In this instance, the sum of the middle two scores is 6. The use of the middle scores helps avoid outliers in the six impairment domains. For instance, if a patient exhibits significant impairment in one area but functions well in their chosen occupation, the median score reflects a balanced assessment. It acknowledges that different domains of impairment are not interchangeable, and the ‘middle’ approach provides a more representative median score.The final PIRS score is derived from Table 14.7 in the AMA Guides.^3^
- Calculate the overall impairment score. The final impairment rating for mental and behavioural disorders is determined by taking the median value of the BPRS, GAF and PIRS scores.^3^

### Writing the report

The examiner is responsible for creating a confidential written report that integrates all available sources of information, both subjective and objective, into a single work product to be shared with the referring party. The report should be organised, complete and comprehensive and should rely on scientifically sound methods for integrating information.

The American Academy of Psychiatry and the Law’s Ethical Guidelines for the Practice of Forensic Psychiatry contain four key ethical principles, namely confidentiality, consent, honesty and striving for objectivity when doing forensic assessments.^29^

A well-crafted report demonstrates qualities such as objectivity, detachment, humanity and professionalism. In contrast, a carelessly constructed report fails to benefit the patient involved, reflects poorly on the author and detracts from the credibility and image of the psychiatry profession.^30^

To write an effective psychiatric IME report, it is important to consider the audience, the intended reader, the purpose of the report and how it will be utilised. Four fundamental principles should be followed^31^:

- Clarity: To ensure the report is understood, formatting should be clear with adequate information, appropriate word choices, good grammar and clear attribution. Formatting recommendations include using a 12-point serif font, maximising white space and numbering the pages.^32^ The correct verb form or tense should be used, such as using past tense for storytelling and present tense for factual information.^33^
- Simplicity: Avoid using complex language sentence structures and jargon. Write in plain and concise language, use the active voice, and keep sentences short. Emotive language should be avoided, and arguments should rely on solid evidence.^34^
- Brevity: Craft a concise report by carefully planning topics and removing irrelevant information during editing. Avoid superfluous words and phrases, and adhere to guidelines for sentence length, paragraph length and section organisation.^34^
- Humanity: Bear in mind that psychiatric reports focus on individuals and their experiences. Incorporate quotations from clients to allow them to speak directly to the reader. Avoid dehumanising language and refer to the person by name with an appropriate title to convey respect.

### Discussion of previous reports

Any discrepancies in the reports perused, the examinee’s account and the examiner’s own findings should be commented on.

### The opinion section

The opinion section is crucial and should address the questions posed in the referral. Use the label ‘Opinion’ to indicate the presentation of professional opinions and ensure that the referral questions are adequately addressed. Any opinions expressed in the report must be based on a synthesis of the best available information rather than on the examiner’s personal belief systems. The primary ethical obligation is to provide a well-reasoned, independent, unbiased, fair, accurate and honest opinion that considers all available evidence. In cases where necessary information is missing, the report should explicitly state this.^8^ Regardless of the nature of the opinion provided, it is important that the report does not appear insensitive to any subjective distress or losses suffered by the examinee.

### Disclaimer section

A statement should be included that explains the opinions and recommendations formed and expressed in the report. The statement should be based on the information available to the writer at the time of the finalisation of the report. The writer reserves the right to change such opinions and recommendations based on the receipt of any additional or updated information.

### Editing

**FIGURE 2:**
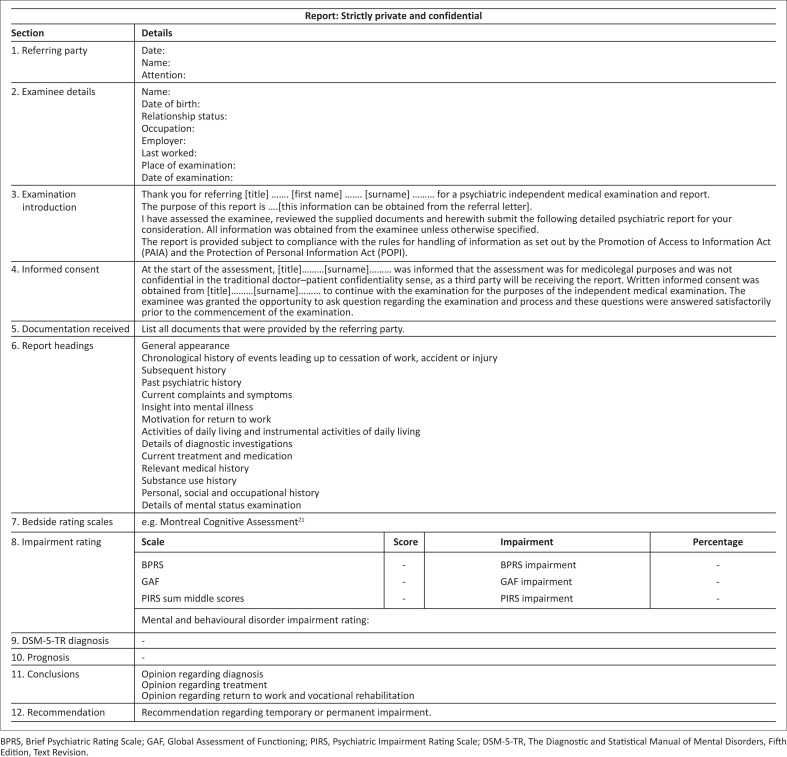
Suggested outline for a psychiatric independent medical examination report.

The organising of the report, the report’s writing style, logical flow and typography should be reviewed and critically evaluated. Common pitfalls should be avoided, such as exaggeration, pompous language, absolute statements, snide comments, pregnant negatives, hedge words, false emphasis and language that makes the report appear impersonal.^30^ A suggested outline for report writing is provided (Figure 2).

By following these recommendations, a well-written psychiatric IME report can effectively communicate the necessary information while maintaining professionalism and respecting the individuals involved.

## Conclusion

In conclusion, the task team drew attention to the importance for both treating and independent psychiatrists in South Africa to adhere to the guidelines outlined in this document. Familiarity with the systematic approach and rating instruments proposed by the AMA Guides to the Evaluation of Permanent Impairment is crucial for adjudication and ensuring fair treatment of mental healthcare users.

The task team emphasised the significance of understanding and addressing the stigma and anxiety often associated with mental health disorders, particularly concerning the IME process. Professionals interacting with individuals undergoing IMEs must possess the necessary understanding, skills and sensitivity to create a supportive environment.

The task team recommended that medical advisors and case managers engage in relevant training courses aligned with these guidelines and procedures to promote continuous improvement. Ongoing professional development will enhance their ability to effectively engage in the IME process and foster a compassionate and empathetic approach towards mental healthcare users.

The guidelines and recommendations presented in this report aim to enhance the quality, credibility and humane nature of the IME process in the field of psychiatry. Implementing these guidelines with conscientiousness will improve accuracy, maintain integrity and affirm the dignity and respect of the examined individuals. By doing so, trust and confidence in the process will be fostered among mental healthcare users and the wider community, ultimately reflecting positively on the profession as a whole.
